# Diuretic and Antidiuretic Activities of Ethanol Extract and Fractions of *Lagopsis supina* in Normal Rats

**DOI:** 10.1155/2019/6927374

**Published:** 2019-12-04

**Authors:** Zhiyong Liu, Li Yang, Longxue Li, Rongrui Wei, Xiaoquan Luo, Tingting Xu, Yun Huang, Zejing Mu, Junwei He

**Affiliations:** ^1^Laboratory Animal Science and Technology Center, Jiangxi University of Traditional Chinese Medicine, Nanchang 330004, China; ^2^Key Laboratory of Modern Preparation of TCM, Ministry of Education, Jiangxi University of Traditional Chinese Medicine, Nanchang 330004, China; ^3^Research Center of Natural Resources of Chinese Medicinal Materials and Ethnic Medicine, Jiangxi University of Traditional Chinese Medicine, Nanchang 330004, China

## Abstract

*Lagopsis supina* is a well-known traditional Chinese medicine and used as an agent for diuresis in China for centuries. This is the first time to evaluate the diuretic activity of the ethanol extract of *L. supina* (LS) and its four fractions (LSA, LSB, LSC, and LSD) in normal rats. After the administration of LS-H, LS-M, LSB-H, and LSC-L, the urine output of the rats was significantly increased, while the urine excretion was significantly reduced after treatment with LSB-L. The urine Na^+^ excretion was remarkably increased with LS-H, LS-M, LSA-H, LSA-L, LSB-H, LSC-L, and LSD-L, and the urine K^+^ excretion was significantly increased after administration of LS-H and LSB-H. Moreover, the urine Na^+^ and K^+^ excretion was significantly reduced after treatment with LSC-H and LSD-H. However, the urine pH values and urine and serum Na^+^-K^+^-ATPase levels did not show remarkable change after administration of LS or its four fractions in comparison with the control group. On the contrary, LS and its four fractions can suppress the renin-angiotensin-aldosterone system (RAAS), including ADH arrest by LSB-H, LSB-L, LSC-L, LSD-L, and LSD-H and ALD arrest by LSD-L, as well as promote ANP release by LS-M, LSB-H, LSC-H, and LSD-H, while furosemide can suppress only arrest of ADH within 24 h compared with the control group. In addition, LS and its four fractions did not change the urine and serum TNF-*α* and IL-6 levels in normal rats within 24 h. This study will provide a quantitative basis for explaining the natural medicinal use of LS as a diuretic agent for edema and promoting the diuretic process.

## 1. Introduction

Natural medicine is considered a quite important resource for drug discovery and has increasingly attracted the attention of researchers [1–4]. In Chinese folk medicine, several traditional Chinese medicines (TCMs) are used as diuretic drugs, but most of them lack pharmacological studies to explain their material basis and its mechanisms [5]. Among them, *Lagopsis supina* (Steph) Ik.-Gal. is a perennial herbaceous plant species of Labiatae family and was widely used as a TCM and known as Xiazhicao (Chinese: 夏至草) [6, 7]. However, due to the indeterminate pharmacological activities and chemical components, *L. supina* still faced big challenges in clinical applications.

Many pharmacological experiments have demonstrated that the crude extract and/or fractions of *L. supina* have promoting blood circulation and removing blood stasis (PBCRBS) [8–10], myocardioprotective [11], and antioxidative [12] effects. Although the pharmacological research of *L. supina* was more, the diuretic activity of LS in normal rats has not yet been studied so far. Furthermore, we found that the ethanol extract of *L. supina* showed a significant diuretic activity in a rat model of traumatic blood stasis (TBS) [13]. However, it is still unclear whether *L. supina*'s diuretic activity is caused by PBCRBS constituents and/or diuretic constituents in the TBS rat model. Therefore, there is an urgent need to figure out the material basis and mechanism of diuretic activity of the ethanol extract and its fractions of *L. supina* in normal rats.

The purpose of the present study was to evaluate the diuretic and antidiuretic activities of the ethanol extract (LS) and its four fractions (LSA, LSB, LSC, and LSD) of *L. supina* in normal rats within 24 h. For the efficacy evaluation, the urine output volume, urinary electrolyte concentrations (Na^+^ and K^+^), and pH values were measured at different time points (1, 2, 4, 6, and 24 h) after administration, and the Na^+^-K^+^-ATPase, angiotensin II (Ang II), atriopeptin (ANP), antidiuretic hormone (ADH), aldosterone (ALD), and TNF-*α* and IL-6 levels in urine and serum were detected within 24 h after administration.

## 2. Materials and Methods

### 2.1. Plant Material and Drug Preparation

The plant material (38.0 kg) and its crude ethanol extract (LS, 8.7 kg, 22.9%) of *L. supina* were reported in our previous paper, where we followed the methods of He et al. [13]. In brief, the whole plants of *L. supina* were collected in Keerqin District, Tongliao City, Inner Mongolia, China, in June 2016 having been identified by Prof. Guoyue Zhong (Jiangxi University of Traditional Chinese Medicine). A voucher specimen (no. XZC201606) was deposited at the Research Center of Natural Resources of Chinese Medicinal Materials and Ethnic Medicine, Jiangxi University of Traditional Chinese Medicine, Nanchang, China. Then, the air-dried and powdered whole plants (38 kg) were exhaustively extracted using 95% EtOH (300 L × 3) and subsequently 50% EtOH (300 L × 3) by maceration at room temperature for seven days. After filtration, combination, and solvent evaporation, the crude ethanol extract of *L. supina* (LS, 8.7 kg, 22.90%) was obtained. Then, LS was suspended in water and extracted with hexane, to afford the hexane portion (LSA, 1320 g) and water portion, respectively. The water portion was chromatographed on a D101 macroporous resin column and eluted with 0%, 30%, 60%, and 95% aqueous EtOH to yield four fractions: 0% (LSB, 6148 g, 16.18%), 30% (LSC, 669 g, 1.76%), 60% (LSD, 260 g, 0.68%), and 95% (LSE, 145 g). The LSA and LSE fractions were combined by high-performance liquid chromatography (HPLC) and thin layer chromatography (TLC) analysis and assigned to LSA (1465 g, 3.86%). The crude ethanol extract and four fractions were resuspended in 0.3% sodium carboxymethyl cellulose (CMC-Na) for subsequent intragastric administration and kept at −20°C until use.

According to clinical practice of TCM [6], the dosage of *L. supina* for rats is 1∼3 g/kg/day, which was reported in our previous paper [13]. In this experiment, the yield of LS was 22.90%, making the dosage of the LS crude extract about 229.0 mg/kg/day.

### 2.2. Chemicals and Reagents

Enzyme-linked immunosorbent assay (ELISA) kits for Na^+^-K^+^-ATPase, Ang II, ANP, ADH, ALD, TNF-*α*, and IL-6 were purchased from Nanjing SenBeiJia Biological Technology Co., Ltd. (Nanjing, China). The levels of urinary Na^+^ and K^+^ were quantified by an automatic biochemical analyzer (OLYMPUS 5421, Olympus Corporation, Japan). All of the other chemicals used in this study were of analytical reagent grade.

### 2.3. Reference Drug

Furosemide (Jiangsu Yabang Aipusen Pharmaceutical Corporation, Jiangsu, China) was used as a reference drug, which was a high-ceiling loop diuretic in clinical practice, and dissolved in water prior to administration.

### 2.4. Animal Treatment and Sample Collection

Male Sprague-Dawley rats weighing 180–220 g were purchased from Hunan SJA Laboratory Animals Co., Ltd. (Changsha, China). All rats were housed in a temperature-controlled room ((23 ± 2)°C) with 12 h light and dark cycles and were allowed free access to food and water ad libitum. All animal procedures were approved by the Institutional Animal Care and Use Committee of Jiangxi University of Traditional Chinese Medicine.

The diuretic activity was determined according to the method published previously [14, 15]. All rats were randomly assigned into 13 groups (*n* = 8 per group): (1) control group (water-loaded rats), (2) furosemide group: 20 mg/kg body weight (BW) of furosemide, (3) LS-L group: 114.5 mg/kg BW of LS, (4) LS-M group: 229.0 mg/kg BW of LS, (5) LS-H group: 458.0 mg/kg BW of LS, (6) LSA-L group: 19.3 mg/kg BW of LSA, (7) LSA-H group: 77.2 mg/kg BW of LSA, (8) LSB-L group: 80.9 mg/kg BW of LSB, (9) LSB-H group: 323.6 mg/kg BW of LSB, (10) LSC-L group: 8.8 mg/kg BW of LSC, (11) LSC-H group: 35.2 mg/kg BW of LSC, (12) LSD-L group: 3.4 mg/kg BW of LSD, and (13) LSD-H group: 13.6 mg/kg BW of LSD. And each group was given the corresponding drug, and the control group received an equivalent amount of water with 0.3% CMC-Na. Each rat was housed individually in a metabolic cage, and the cumulative urine output was measured at 1, 2, 4, 6, and 24 h after administration. Urinary electrolyte concentrations (Na^+^ and K^+^) and pH values were determined in the 24 h urine samples of all rats. The blood samples were collected from the abdominal aorta of rats and subsequently subjected to centrifugation (3000 rpm at 4°C for 10 min). The urine and serum were stored at −20 and −80°C for further analysis, respectively.

### 2.5. Biochemical Methods

Levels of Na^+^-K^+^-ATPase, Ang II, ANP, ADH, ALD, TNF-*α*, and IL-6 in urine and serum were determined using commercial ELISA kits (Nanjing SenBeiJia Biological Technology Co., Ltd., Nanjing, China), which were performed as per the manufacturer's instructions.

### 2.6. Statistical Analysis

Data were analyzed using SPSS Statistics V17.0 software and presented as mean ± standard deviation (SD). The differences between the groups were assessed by a one-way analysis of variance (ANOVA) and Tukey's test. Differences with *P* < 0.05 indicated statistical significance.

## 3. Results

### 3.1. Effects on Urinary Excretion Volume

To determine whether the ethanol extract and its four fractions (LSA, LSB, LSC, and LSD) of LS have the diuretic effects, we measured the urinary excretion volume in every group at different time points (1, 2, 4, 6, and 24 h) after treatment with LS and its four fractions or furosemide. The urine output volume increased significantly (*P* < 0.05 or *P* < 0.01) for LSA-L (19.3 mg/kg), LS-L (114.5 mg/kg), and LS-M (229.0 mg/kg) within 1, 2, and 4 h, respectively, while LSD-H (13.6 mg/kg) produced a remarkable reduction in the urinary excretion volume within 4 h after administration compared with the control group (*P* < 0.05, Table 1). In addition, urinary excretion volume in LS-H (458.0 mg/kg), LS-M (229.0 mg/kg), LSB-H (322.6 mg/kg), and LSC-L (8.8 mg/kg) groups significantly increased within 24 h after administration (*P* < 0.05 or *P* < 0.01), while LSB-L (80.9 mg/kg) produced a remarkable reduction in the urinary excretion volume within 24 h after administration (*P* < 0.01, Table 1). Actually, LSA-H (77.2 mg/kg), LSC-H (35.2 mg/kg), LSD-H (13.6 mg/kg), and LSD-L (3.4 mg/kg) also contributed to urine output volume, and the increase did not reach statistical significance within 24 h after administration.

Compared with that in the control group, the urine output volume in the furosemide group significantly increased within 1, 2, 4, 6, and 24 h after administration (*P* < 0.01, Table 1). Moreover, the urine output volume in the furosemide group was significantly increased compared with that in other groups within 1, 2, 4, and 6 h after administration (*P* < 0.01, Table 1); however, the cumulative urine output volume in the furosemide group and LSB-H group within 24 h showed no significant difference (Table 1).

### 3.2. Effects on Urinary pH

As shown in Table 2, LS and its four fractions did not change the urine pH in normal rats within 24 h in comparison with the control group. The results indicated that LS and its four fractions might play major roles in acid-basic balances in normal rats within 24 h.

### 3.3. Effects on Urinary Electrolyte Concentrations

We also examined urine Na^+^ and K^+^ concentrations in every group at different time points (1, 2, 4, 6, and 24 h) after treatment with LS and its four fractions or furosemide (Tables 3 and 4).

Compared with that in the normal group, the urine Na^+^ concentration in the furosemide treatment group was significantly reduced within 4 h, while the Na^+^ concentration was significantly increased within 24 h (*P* < 0.05 or *P* < 0.01, Table 3). In contrast, the urine K^+^ concentration was remarkably increased in the furosemide group within 6 h, while the K^+^ concentration was dramatically reduced within 24 h (*P* < 0.05 or *P* < 0.01, Table 4).

After the oral administration of LS and its four fractions within 24 h, a great variation was also observed in urinary electrolyte Na^+^ and K^+^ concentrations. Administration of LS-H (458.0 mg/kg), LS-M (229.0 mg/kg), LSA-H (77.2 mg/kg), LSA-L (19.3 mg/kg), LSB-H (322.6 mg/kg), LSC-L (8.8 mg/kg), and LSD-L (3.4 mg/kg) significantly increased the urinary electrolyte Na^+^ concentrations within 24 h (*P* < 0.05 or *P* < 0.01), while LSC-H (35.2 mg/kg) and LSD-H (13.6 mg/kg) produced a remarkable reduction in the urinary electrolyte Na^+^ concentrations within 24 h (*P* < 0.05 or *P* < 0.01, Table 3). Furthermore, the levels of K^+^ concentrations were remarkably reduced after treatment with oral administration of LSC-H (35.2 mg/kg) and LSD-H (13.6 mg/kg), while LS-H (458.0 mg/kg) and LSB-H (323.6 mg/kg) significantly increased the urinary electrolyte K^+^ concentrations within 24 h (*P* < 0.05 or *P* < 0.01, Table 4).

### 3.4. Effects on Urine and Serum Levels of Na^+^-K^+^-ATPase

Since conventional diuretics can induce electrolyte changes in urine and serum, we examined the levels of Na^+^-K^+^-ATPase in urine and serum of normal rats and drug-treated rats. However, administration of LS and its four fractions did not significantly change the urine and serum Na^+^-K^+^-ATPase levels in normal rats within 24 h in comparison with the control group (Table 5).

### 3.5. Effects on Urine and Serum Levels of Ang II, ANP, ADH, and ALD

As shown in Tables 6 and 7, the urine levels of ADH were remarkably reduced after treatment with oral administration of furosemide within 24 h (*P* < 0.01). With the treatment of LS-M (229.0 mg/kg), the levels of ANP in urine were significantly increased within 24 h (*P* < 0.05).

Interestingly, the four fractions of LS remarkably reduced ADH and ALD levels and significantly increased ANP levels in urine and/or serum compared with control group rats within 24 h (*P* < 0.05 or *P* < 0.01, Tables 6 and 7). Treatment with LSB tended to increase ANP levels and to reduce ADH levels in urine compared with control group rats within 24 h (*P* < 0.01). Moreover, the levels of ADH reduced and those of ANP increased in urine and/or serum significantly after treatment with LSC compared with control group rats within 24 h (*P* < 0.05 or *P* < 0.01). In addition, we also found that the levels of ADH and ALD were reduced and ANP levels were increased in urine and/or serum significantly after treatment with LSD compared with control group rats within 24 h (*P* < 0.05 or *P* < 0.01).

The result indicates that LS and its four fractions can promote ANP release and suppress the RAAS, including the arrest of ADH and ALD, while furosemide can suppress only arrest of ADH. Thus, as a diuretic, LS and its fractions are superior to furosemide based on multiple components, multiple channels, and multiple targets to treat diuretic diseases.

### 3.6. Effects on Urine and Serum Levels of TNF-*α* and IL-6

Inflammation is a complex biological response of the innate immune system and produces a number of proinflammatory mediators, such as TNF-*α*, IL-6, and others. The levels of TNF-*α* and IL-6 in urine and serum were measured to assess the effect of LS and its four fractions on inflammation by respective ELISA kits. As shown in Table 8, the ELISA results revealed that administration of LS and its four fractions did not affect the urine and serum TNF-*α* and IL-6 levels in normal rats within 24 h in comparison with the control group.

## 4. Discussion

The urinary excretion volume was mainly determined by the urine concentrating ability, including urine and its electrolyte reabsorption [13, 16]. In this study, we explored the diuretic activity of LS and its four fractions (LSA, LSB, LSC, and LSD) compared with that produced by furosemide in normal rats. After oral administration of LS and its four fractions, LS-H (458.0 mg/kg), LS-M (229.0 mg/kg), LSB-H (323.6 mg/kg), and LSC-L (8.8 mg/kg) produced notable diuretic effects in comparison with the control group within 24 h. It is important that the 323.6 mg/kg dose of LSB-H significantly increased the urine output volume comparable to furosemide within 24 h. Surprisingly, we determined that LSB has a dual effect on renal function, including promoting diuretic activity at a higher dose (323.6 mg/kg) and inhibiting diuretic activity at a lower dose (80.9 mg/kg), which is similar to other diuretics in TCM [14, 15]. On the contrary, our results demonstrate a preliminary linear relationship between the LS and LSB doses and the diuretic biological response. Further research is underway to elucidate the dose-diuretic activity relationship of LS and its four fractions in normal rats. These results indicated that LS, LSB, and LSC present a notable diuretic effect, and the LSB dose should be given more attention in clinical applications.

Na^+^ and K^+^ were the major electrolytes located in the intracellular and extracellular fluids and play a regulatory role in maintaining body fluid homeostasis [17–19]. Our results showed that LS and its four fractions affect the urinary electrolytes and the acid-base alterations compared with the control group within 24 h in normal rats. A great variation in electrolyte excretion (Na^+^ and K^+^) between promoting diuretic effects and inhibiting diuretic effects of LS and its four fractions as well as furosemide were observed. When promoting the urine output, LS, LSA, LSB, LSC, LSD, and furosemide produced similar increases in urine Na^+^ excretion but had slight effects on K^+^ excretion. This increased Na^+^ loss, which is due to the increase in the distal tubular K^+^ concentration, and stimulated the ALD-sensitive potassium pump to increase the K^+^ reabsorption in exchange for Na^+^ and H^+^, which were excreted into the urine [15, 20]. Although the LS and its four fractions affected the Na^+^ and K^+^ concentrations in urine, all drug groups cannot affect urine or serum Na^+^-K^+^-ATPase levels and urine pH values compared with the control group within 24 h.

The RAAS is very important for body fluid regulation and plays a prominent role in controlling fluid homeostasis and maintaining electrolyte balance [18, 21, 22]. Moreover, the main three hormones in the RAAS including Ang II, ADH, and ALD have been reported to decrease the glomerular filtration rate and increase tubular reabsorption of sodium ions [16–18]. ANP not only increases diuresis and Na^+^ electrolyte excretion but also inhibits the activation of RAAS and blocks the release and/or actions of ADH and ALD [23–27]. On the contrary, ALD promotes urine Na^+^ and H_2_O reabsorption and regulates Ang II and ALD levels [18]. In addition, Ang II could also augment Na^+^ reabsorption and the release of ADH. In this experiment, LS and its four fractions can suppress the RAAS, including ADH arrest by LSB (80.9 and 323.6 mg/kg), LSC (8.8 mg/kg), and LSD (3.4 and 13.6 mg/kg) and ALD arrest by LSD (3.4 mg/kg), as well as promote ANP release by LS (229.0 mg/kg), LSB (323.6 mg/kg), LSC (35.2 mg/kg), and LSD (13.6 mg/kg), while furosemide can suppress only arrest of ADH within 24 h compared with the control group. These results indicated that the diuretic effect of LS and its four fractions on normal rats is multichannel and multitarget including ANP, ADH, and ALD. Meanwhile, furosemide only affects the ADH level.

Therefore, we believe that LS and its active components are involved in the treatment of diuretic diseases with multiple components, multiple channels, and multiple targets. Although the diuretic and antidiuretic effects of these active components are not perfectly elucidated, previous phytochemical investigations suggested that diterpenoids, phenylethanoids, and flavonoids are their major components and exhibit various bioactivities [28–30]. Based on the above data, we hypothesized that these major bioactive components are responsible for the diuretic and antidiuretic activities, but much work still needs to be done.

Inflammation is a complex biological response to harmful stimuli, tissue injury, or infection and produced a number of proinflammatory mediators, such as TNF-*α* and IL-6 [31, 32]. Moreover, inflammatory cytokines, such as IL-6 and TNF, are involved in the processes by which TCMs act on PBCRBS according to experimental researches and network pharmacology analysis [33–35]. On the contrary, the traditional Chinese medical concept of *Huo Xue Li Shui* (Chinese: 活血利水) refers to “PBCRBS” and “promoting blood circulation and diuresis” (PBCD), which excretes H_2_O out of the body in the form of urine [13]. Our previous study showed that the ethanol extract of *L. supina* showed a significant diuretic activity in TBS model rats, which are a rat model for the screening of PBCRBS and diuretic drugs [13]. For this reason, we also examined the levels of TNF-*α* and IL-6 in rats after treatment with LS and its four fractions (LSA-D) in this experiment. The ELISA results revealed that administration of LS and its four fractions did not affect the urine and serum TNF-*α* and IL-6 levels in normal rats within 24 h in comparison with the control group. These results suggested that LS and its four fractions did not affect the innate immune system in normal rats.

## 5. Conclusions

To summarize our findings, this is the first reported notable diuretic effect of LS and its fractions (LSB and LSC) in normal rats, which probably inhibit the RAAS system and promote ANP release through multiple channels and multiple targets. We also first found that LSB has dual effects, namely, promoting and inhibiting diuretics in normal rats. Although the LS and its four fractions could change the urinary electrolyte concentrations (Na^+^ and K^+^), they did not affect the urine pH and urine and serum Na^+^-K^+^-ATPase, TNF-*α*, and IL-6 levels within 24 h compared with the control group. This study will provide a quantitative basis for explaining the natural medicinal use of LS as a diuretic agent for edema and promoting the diuretic process. Further research is underway to elucidate the mechanism and determine the active components responsible for the diuretic and antidiuretic activities of LS.

## Figures and Tables

**Table 1:** Effects of LS, LSA, LSB, LSC, and LSD on urinary excretion volume (mL) within 1, 2, 4, 6, and 24 h after oral administration.

Group	1 h	2 h	4 h	6 h	24 h
Control	2.07 ± 0.64	4.45 ± 1.28	6.77 ± 1.23	9.13 ± 1.25	17.73 ± 0.80
Furosemide	7.15 ± 2.00^*∗∗*^	11.45 ± 1.69^*∗∗*^	14.02 ± 2.11^*∗∗*^	16.85 ± 1.89^*∗∗*^	34.68 ± 3.51^*∗∗*^
LS-H	2.37 ± 1.00	4.53 ± 2.08	6.58 ± 2.22	9.72 ± 2.28	28.27 ± 3.89^*∗∗*^
LS-M	3.28 ± 1.64	6.22 ± 1.97	8.87 ± 1.65^*∗*^	10.63 ± 1.79	25.30 ± 2.71^*∗∗*^
LS-L	2.98 ± 1.05	6.52 ± 1.63^*∗*^	8.18 ± 1.60	9.43 ± 1.56	17.17 ± 1.95
LSA-H	3.50 ± 1.47	6.27 ± 2.21	8.27 ± 2.08	10.53 ± 2.30	24.68 ± 2.50
LSA-L	3.98 ± 0.89^*∗∗*^	5.80 ± 1.45	7.37 ± 1.75	9.45 ± 1.98	19.75 ± 2.07
LSB-H	2.78 ± 1.36	5.30 ± 1.38	7.82 ± 1.60	10.98 ± 2.08	38.18 ± 3.45^*∗∗*^
LSB-L	3.12 ± 0.99	5.18 ± 1.37	6.63 ± 1.76	8.68 ± 1.02	15.13 ± 1.97^*∗∗*^
LSC-H	2.00 ± 0.93	5.00 ± 1.74	6.14 ± 2.01	8.10 ± 1.07	24.62 ± 3.07
LSC-L	2.98 ± 0.81	5.52 ± 0.96	7.83 ± 1.97	12.27 ± 1.41	27.25 ± 3.00^*∗∗*^
LSD-H	2.25 ± 1.00	3.77 ± 1.23	5.10 ± 1.11^*∗*^	7.33 ± 1.81	20.73 ± 2.80
LSD-L	1.19 ± 0.61	4.08 ± 0.90	6.58 ± 1.41	9.23 ± 1.74	21.02 ± 2.38

The cumulative values are reported as mean ± SD for 8 rats in each group. ^*∗*^*P* < 0.05 and ^*∗∗*^*P* < 0.01 compared to the control.

**Table 2:** Effects of LS, LSA, LSB, LSC, and LSD on urinary pH within 1, 2, 4, 6, and 24 h after oral administration.

Group	1 h	2 h	4 h	6 h	24 h
Control	7.48 ± 0.46	7.39 ± 0.54	7.71 ± 0.19	7.79 ± 0.16	7.58 ± 0.72
Furosemide	7.66 ± 0.66	7.59 ± 0.67	7.47 ± 0.50	7.21 ± 0.74	7.69 ± 0.84
LS-H	7.67 ± 0.43	7.65 ± 0.35	7.43 ± 0.48	7.51 ± 0.48	8.08 ± 0.21
LS-M	7.98 ± 0.49	7.68 ± 0.75	7.77 ± 0.72	7.68 ± 0.91	8.04 ± 0.63
LS-L	7.86 ± 0.28	7.83 ± 0.43	7.73 ± 0.68	7.88 ± 0.56	8.13 ± 0.70
LSA-H	7.64 ± 0.63	7.74 ± 0.62	7.57 ± 0.61	7.43 ± 0.78	8.18 ± 0.50
LSA-L	7.81 ± 0.42	7.57 ± 0.46	7.31 ± 0.33	7.41 ± 0.50	8.13 ± 0.51
LSB-H	7.74 ± 0.38	7.60 ± 0.37	7.58 ± 0.30	7.40 ± 0.38	7.84 ± 0.65
LSB-L	7.83 ± 0.40	7.78 ± 0.38	7.76 ± 0.63	7.64 ± 0.54	8.42 ± 0.56
LSC-H	7.68 ± 0.27	7.50 ± 0.51	7.54 ± 0.59	7.58 ± 0.51	7.97 ± 0.24
LSC-L	7.72 ± 0.56	7.63 ± 0.51	7.39 ± 0.54	7.58 ± 0.51	8.16 ± 0.37
LSD-H	7.64 ± 0.64	7.81 ± 0.70	7.94 ± 0.74	7.80 ± 0.85	8.26 ± 0.57
LSD-L	7.83 ± 0.45	7.69 ± 0.46	7.35 ± 0.35	7.08 ± 0.44	8.25 ± 0.61

The values are reported as mean ± SD for 8 rats in each group.

**Table 3:** Effects of LS, LSA, LSB, LSC, and LSD on urinary electrolyte Na^+^ (mmol/L) concentrations within 1, 2, 4, 6, and 24 h after oral administration.

Group	1 h	2 h	4 h	6 h	24 h
Control	101.88 ± 7.77	100.38 ± 8.50	99.33 ± 9.33	100.18 ± 2.04	100.30 ± 3.87
Furosemide	59.75 ± 1.34^*∗∗*^	67.43 ± 8.02^*∗∗*^	84.65 ± 3.77^*∗*^	96.00 ± 5.53	111.80 ± 8.59^*∗*^
LS-H	111.80 ± 8.59	114.13 ± 8.26	117.45 ± 8.13^*∗*^	120.15 ± 7.29^*∗∗*^	119.80 ± 8.69^*∗∗*^
LS-M	101.55 ± 11.09	102.28 ± 9.82	107.48 ± 9.73	114.15 ± 11.83	120.68 ± 1.78^*∗∗*^
LS-L	118.20 ± 6.20^*∗*^	117.30 ± 7.47^*∗*^	116.58 ± 6.93^*∗*^	115.83 ± 6.51^*∗∗*^	108.20 ± 10.16
LSA-H	114.95 ± 18.99	116.13 ± 14.65	121.00 ± 17.08	125.00 ± 12.53	142.28 ± 18.97^*∗∗*^
LSA-L	75.98 ± 7.60^*∗*^	78.00 ± 4.45^*∗*^	91.18 ± 6.00	100.28 ± 6.75	121.70 ± 9.37^*∗∗*^
LSB-H	128.18 ± 2.54^*∗∗*^	124.05 ± 3.26^*∗∗*^	111.65 ± 5.31	107.83 ± 4.08^*∗*^	111.55 ± 4.57^*∗∗*^
LSB-L	115.78 ± 9.47	116.95 ± 17.14	109.33 ± 15.01	102.30 ± 11.55	91.00 ± 7.95
LSC-H	94.95 ± 4.84	94.90 ± 5.96	85.10 ± 7.54	83.05 ± 9.22^*∗*^	78.38 ± 8.23^*∗∗*^
LSC-L	114.73 ± 9.48	110.80 ± 7.39	111.38 ± 11.17	112.35 ± 6.18^*∗∗*^	126.13 ± 10.13^*∗∗*^
LSD-H	106.03 ± 7.42	98.60 ± 4.03	94.50 ± 3.89	88.10 ± 4.62^*∗∗*^	74.43 ± 7.09^*∗∗*^
LSD-L	115.55 ± 5.26^*∗*^	113.10 ± 4.52^*∗*^	107.33 ± 5.44	112.08 ± 2.39^*∗∗*^	118.63 ± 5.80^*∗∗*^

The values are reported as mean ± SD for 8 rats in each group. ^*∗*^*P* < 0.05 and ^*∗∗*^*P* < 0.01 compared to the control.

**Table 4:** Effects of LS, LSA, LSB, LSC, and LSD on urinary electrolyte K^+^ (mmol/L) concentrations within 1, 2, 4, 6, and 24 h after oral administration.

Group	1 h	2 h	4 h	6 h	24 h
Control	78.98 ± 5.35	79.40 ± 5.53	79.38 ± 5.41	78.83 ± 5.08	79.03 ± 5.38
Furosemide	150.85 ± 26.84^*∗∗*^	148.80 ± 24.93^*∗∗*^	122.00 ± 10.53^*∗∗*^	95.20 ± 1.40^*∗∗*^	61.55 ± 10.50^*∗*^
LS-H	97.80 ± 2.37^*∗∗*^	96.88 ± 2.29^*∗∗*^	95.35 ± 2.03^*∗∗*^	94.85 ± 2.33^*∗*^	94.48 ± 2.92^*∗∗*^
LS-M	93.83 ± 6.13^*∗*^	91.28 ± 5.01^*∗*^	85.73 ± 4.81	84.00 ± 5.36	85.38 ± 4.60
LS-L	86.80 ± 6.34	85.45 ± 6.12	85.43 ± 3.75	84.00 ± 3.41	74.88 ± 6.97
LSA-H	62.18 ± 3.20^*∗∗*^	58.20 ± 3.40^*∗∗*^	57.30 ± 2.08^*∗∗*^	61.25 ± 0.94^*∗∗*^	77.50 ± 5.12
LSA-L	76.28 ± 7.20	75.83 ± 6.19	77.58 ± 4.64	77.75 ± 4.48	76.75 ± 2.86
LSB-H	79.75 ± 2.41	81.08 ± 1.86	81.98 ± 2.75	82.20 ± 2.82	90.28 ± 5.46^*∗*^
LSB-L	65.50 ± 11.12	68.33 ± 7.23	68.58 ± 7.01	68.83 ± 7.47	71.58 ± 8.31
LSC-H	70.43 ± 3.19^*∗*^	69.53 ± 2.28^*∗*^	69.63 ± 1.79^*∗*^	67.68 ± 2.34^*∗∗*^	58.78 ± 5.93^*∗∗*^
LSC-L	82.15 ± 10.62	81.33 ± 10.07	79.80 ± 9.77	74.53 ± 8.26	75.80 ± 10.62
LSD-H	65.25 ± 5.55^*∗*^	63.38 ± 5.58^*∗∗*^	61.85 ± 4.26^*∗∗*^	60.58 ± 4.03^*∗∗*^	52.13 ± 6.36^*∗∗*^
LSD-L	85.78 ± 3.46	83.23 ± 2.10	81.28 ± 2.99	80.48 ± 1.50	78.30 ± 1.26

The values are reported as mean ± SD for 8 rats in each group. ^*∗*^*P* < 0.05 and ^*∗∗*^*P* < 0.01 compared to the control.

**Table 5:** Effects of LS, LSA, LSB, LSC, and LSD on the levels of Na^+^-K^+^-ATPase (U/L) in rat urine and serum within 24 hours after oral administration.

Group	Urine	Serum
Control	92.95 ± 16.67	105.21 ± 16.41
Furosemide	100.3 ± 16.10	107.42 ± 13.66
LS-H	99.35 ± 19.76	90.41 ± 15.82
LS-M	116.4 ± 17.37	90.72 ± 10.28
LS-L	87.29 ± 14.46	99.99 ± 10.65
LSA-H	99.71 ± 15.73	89.25 ± 16.40
LSA-L	95.68 ± 18.08	91.72 ± 14.58
LSB-H	94.54 ± 12.40	98.32 ± 19.97
LSB-L	106.55 ± 11.51	102.66 ± 15.34
LSC-H	114.59 ± 9.68	116.97 ± 17.48
LSC-L	114.93 ± 15.05	96.21 ± 19.63
LSD-H	112.65 ± 15.58	87.12 ± 10.88
LSD-L	102.82 ± 13.12	106.06 ± 13.50

The values are reported as mean ± SD for 8 rats in each group.

**Table 6:** Effects of LS, LSA, LSB, LSC, and LSD on the levels of Ang II, ANP, ADH, and ALD (pg/mL) in rat urine within 24 h after oral administration.

Group	Ang II	ANP	ADH	ALD
Control	215.7 ± 16.76	199.69 ± 13.48	24.46 ± 1.42	236.03 ± 16.08
Furosemide	197.30 ± 14.71	223.41 ± 18.81	18.42 ± 2.57^*∗∗*^	205.60 ± 12.87
LS-H	206.79 ± 18.88	229.88 ± 11.52	22.75 ± 2.04	200.7 ± 15.40
LS-M	246.53 ± 18.14	199.61 ± 14.38	21.16 ± 1.66	205.56 ± 14.94
LS-L	218.32 ± 11.81	188.30 ± 17.64	24.69 ± 1.50	214.25 ± 14.94
LSA-H	238.72 ± 16.79	227.79 ± 19.81	23.12 ± 1.92	188.04 ± 13.13
LSA-L	201.67 ± 10.33	208.45 ± 15.71	23.66 ± 1.88	211.11 ± 17.39
LSB-H	176.96 ± 10.49	234.5 ± 8.07^*∗∗*^	20.74 ± 1.97^*∗∗*^	254.52 ± 13.87
LSB-L	218.62 ± 17.33	207.66 ± 10.41	18.58 ± 1.11^*∗∗*^	196.14 ± 14.19
LSC-H	219.44 ± 10.70	213.31 ± 10.31	21.49 ± 1.48	221.32 ± 11.41
LSC-L	235.24 ± 13.08	211.69 ± 10.58	20.00 ± 1.39^*∗∗*^	204.36 ± 14.31
LSD-H	223.88 ± 10.96	212.51 ± 13.61	20.28 ± 1.15^*∗*^	202.58 ± 10.65
LSD-L	210.98 ± 16.70	227.27 ± 17.80^*∗*^	20.74 ± 2.02	182.02 ± 16.32^*∗*^

The values are reported as mean ± SD for 8 rats in each group. ^*∗*^*P* < 0.05 and ^*∗∗*^*P* < 0.01 compared to the control.

**Table 7:** Effects of LS, LSA, LSB, LSC, and LSD on the levels of Ang II, ANP, ADH, and ALD (pg/mL) in rat serum within 24 h after oral administration.

Group	Ang II	ANP	ADH	ALD
Control	219.43 ± 16.19	187.47 ± 12.00	23.09 ± 1.12	229.78 ± 15.03
Furosemide	218.53 ± 15.99	186.85 ± 12.83	20.31 ± 1.70	193.19 ± 15.65
LS-H	219.88 ± 10.81	217.13 ± 14.22^*∗*^	20.11 ± 1.11	232.80 ± 19.79
LS-M	213.24 ± 10.54	232.36 ± 15.83	23.70 ± 1.83	222.98 ± 18.29
LS-L	199.66 ± 13.47	219.78 ± 11.54	20.54 ± 1.28	202.21 ± 19.17
LSA-H	234.87 ± 11.86	193.99 ± 9.21	20.32 ± 1.01	231.86 ± 14.03
LSA-L	240.34 ± 14.86	231.18 ± 13.70	20.30 ± 1.10	171.40 ± 5.83
LSB-H	253.99 ± 13.11	224.45 ± 17.83	19.59 ± 1.99	201.52 ± 14.85
LSB-L	239.09 ± 17.55	202.17 ± 19.57	21.43 ± 1.64	216.85 ± 17.01
LSC-H	215.55 ± 11.47	211.94 ± 3.83^*∗*^	19.93 ± 1.39	229.79 ± 14.31
LSC-L	235.24 ± 12.85	213.27 ± 16.63	22.70 ± 1.97	209.30 ± 18.69
LSD-H	203.79 ± 15.93	205.46 ± 14.23	17.09 ± 0.66^*∗∗*^	200.22 ± 8.46
LSD-L	231.49 ± 16.22	206.85 ± 19.71	19.50 ± 1.84^*∗*^	186.51 ± 18.13^*∗*^

The values are reported as mean ± SD for 8 rats in each group. ^*∗*^*P* < 0.05 and ^*∗∗*^*P* < 0.01 compared to the control.

**Table 8:** Effects of LS, LSA, LSB, LSC, and LSD on TNF-*α* and IL-6 levels in urine and serum of rats within 24 hours after administration.

Group	Urine		Serum	
	TNF-*α*	IL-6	TNF-*α*	IL-6
Control	145.23 ± 22.52	82.98 ± 13.99	143.26 ± 28.06	72.33 ± 14.42
Furosemide	143.63 ± 27.57	72.49 ± 13.88	157.04 ± 18.19	73.00 ± 13.80
LS-H	124.22 ± 13.81	74.28 ± 15.85	132.04 ± 29.98	65.00 ± 16.58
LS-M	133.71 ± 10.65	71.04 ± 15.41	139.90 ± 26.89	68.20 ± 13.81
LS-L	140.29 ± 12.81	78.68 ± 16.32	144.14 ± 14.82	69.83 ± 16.67
LSA-H	129.00 ± 16.04	72.29 ± 11.08	135.28 ± 30.58	74.12 ± 12.77
LSA-L	150.42 ± 21.77	66.29 ± 13.32	125.14 ± 12.81	70.25 ± 11.50
LSB-H	142.03 ± 15.04	78.49 ± 13.28	152.62 ± 28.04	69.88 ± 13.63
LSB-L	133.51 ± 10.80	69.42 ± 13.98	139.21 ± 31.27	71.76 ± 16.86
LSC-H	131.41 ± 21.70	69.89 ± 12.69	146.63 ± 24.64	62.38 ± 11.05
LSC-L	130.78 ± 19.43	61.23 ± 12.00	150.14 ± 14.18	73.59 ± 12.98
LSD-H	146.98 ± 23.31	71.45 ± 6.88	139.26 ± 21.28	73.82 ± 19.00
LSD-L	140.21 ± 19.62	81.43 ± 14.91	134.78 ± 32.01	75.81 ± 15.07

The values are reported as mean ± SD for 8 rats in each group.

## Data Availability

The data used to support the findings of this study are available from the corresponding author upon request.

## Abbreviations

LS: The ethanol extract of *Lagopsis supina*
RAAS: Renin-angiotensin-aldosterone system
TCMs: Traditional Chinese medicines
PBCRBS: Promoting blood circulation and removing blood stasis
TBS: Traumatic blood stasis
Ang II: Angiotensin II
ANP: Atriopeptin
ADH: Antidiuretic hormone
ALD: Aldosterone
HPLC: High-performance liquid chromatography
TLC: Thin layer chromatography
CMC-Na: Sodium carboxymethyl cellulose
ELISA: Enzyme-linked immunosorbent assay
SD: Standard deviation
ANOVA: Analysis of variance
TNF: Tumor necrosis factor
IL-6: Interleukin-6.
